# Application of a buprenorphine transdermal patch for the perioperative analgesia in patients who underwent simple lumbar discectomy

**DOI:** 10.1097/MD.0000000000006844

**Published:** 2017-05-19

**Authors:** Jian Tang, Jin Fan, Yilun Yao, Weihua Cai, Guoyong Yin, Wei Zhou

**Affiliations:** Department of Orthopaedic, The First Affiliated Hospital of Nanjing Medical University, Nanjing, China.

**Keywords:** analgesia, buprenorphine transdermal patch, lumbar disk herniation, perioperative period

## Abstract

This study aimed to investigate the perioperative analgesic effect of a buprenorphine transdermal patch in patients who underwent simple lumbar discectomy.

In total, 96 patients were randomly divided into parecoxib intravenous injection (Group A), oral celecoxib (Group B), and buprenorphine transdermal patch groups (Group C). The pain status, degree of satisfaction, adverse effects, and condition in which the patient received tramadol hydrochloride for uncontrolled pain were recorded on the night before surgery, postoperative day 1, postoperative day 3, and postoperative day 5.

The degree of patient satisfaction in Group C was higher than that in Groups A and B, with minimal adverse effects.

The buprenorphine transdermal patch had a better perioperative analgesic effect in patients who underwent simple lumbar discectomy.

## 1 Introduction

The constitution of the population has changed, and the pace of modern life has rapidly increased with the advancement of society. Moreover, a significant increase in the incidence of lumbar disk herniation (LDH) has been observed. LDH often attacks young adults and is the most common cause of low back and leg pain.^[1,2]^ Due to its simple operation, convenience, less trauma, and definite curative effect, simple discectomy has become one of the main surgical strategies for treating LDH due to its simple procedure, convenience, less trauma, and definitive curative effect. It has been widely applied in clinical practice.^[3]^ However, the postoperative acute pain seriously affects the postoperative physical and psychological status and functional rehabilitation.^[4]^ Effective postoperative analgesia not only is beneficial to the functional recovery in patients, but also could improve the degree of patient satisfaction, minimize hospitalization time, and reduce the occurrence of related complications, such as thrombosis and so on.^[5]^ Nowadays, in clinical practice, the main analgesic methods include oral analgesics, local analgesics administered through an intramuscular or intravenous injection, and intraspinal analgesics. Each analgesic method has its own advantages and disadvantages. The buprenorphine transdermal patch is a kind of transdermal patch containing the analgesic drug (buprenorphine), which facilitates continuous drug release at a steady pace. The analgesic drug is percutaneously absorbed and enters into the circulatory system. The buprenorphine transdermal patch has a higher bioavailability and can achieve a more stable concentration in the blood, hence exerting a better analgesic effect. It has been widely applied in treating chronic low back pain abroad, displaying excellent results.^[6–9]^ Since March 2014, some patients who underwent simple lumbar discectomy in the Jiangsu Province Hospital were administered a buprenorphine transdermal patch (preemptive analgesia regimen) to alleviate perioperative pain. This study aimed to investigate the analgesic effect and safety of a buprenorphine transdermal patch in clinical practice, compared with the conventional analgesic regimen of parecoxib intravenous injection and oral celecoxib, to provide new insights for painless ward management in an orthopedic unit.

## 2 Material and methods

### 2.1 General data

The study was randomized controlled study and approved by the severance Institutional Review Board of the First Affiliated Hospital with Nanjin Medical University. A total of 96 patients (55 males and 41 females), who underwent simple discectomy under general anesthesia for treating LDH, were enrolled in the study between March 2014 and December 2015. The patients were randomly divided into parecoxib intravenous injection (Group A), oral celecoxib (Group B), and buprenorphine transdermal patch groups (Group C) (32 patients in each group). No significant difference in age, gender, body weight, preoperative visual analog scale (VAS) score, and surgical time was found between the groups (*P* > .05). The results are shown in Table 1.

**Table 1:**
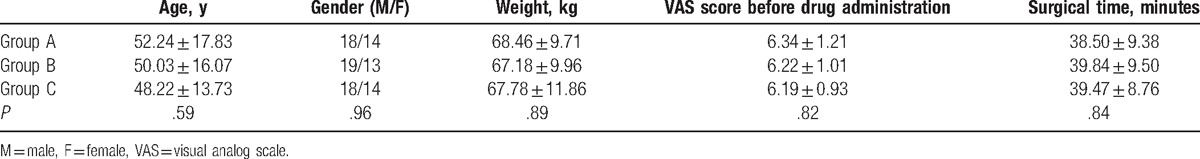
General data of the patients in the 3 groups.

The inclusion criteria were as follows: (1) patients who underwent single-segment LDH; patients aged between 18 years and 75 years; patients with American Society of Anesthesiologists grades I and II; patients who underwent simple lumbar discectomy; and patients who signed informed consent and agreed to participate in this study. The exclusion criteria were as follows: (1) patients having a medical history of cardiopulmonary diseases, cerebrovascular disease, and liver and kidney dysfunction; (2) patients with active peptic ulcer or gastrointestinal bleeding or mental disorder; (3) patients with a long-term medication history of opioid and nonopioid analgesics; (4) patients having neuromuscular disease in the affected limb; and (5) patients having drug allergy for any of the aforementioned analgesic drugs. All the patients were treated by the physicians of the same medical group. The patients were administered general anesthesia. During the surgery, the patients were administered anesthetics of the same category. The study protocol was designed in accordance with the Declaration of Helsinki guidelines and approved by the Medical Ethics Committee of the hospital. All subjects provided a written informed consent form prior to participation. All patients received preoperative health education.

### 2.2 Surgical process

All the enrolled patients were treated with simple lumbar discectomy. A median longitudinal incision was made, and the intervertebral disk of the lesion segment was exposed under general anesthesia. A part of the adjacent vertebral plate could be moved upward and downward. The ligamentum flavum was resected, the dural sac and nerve root were revealed, and the nerve root was separated. The intervertebral disk and vertebral pulp were exposed, and the annulus fibrosus was then cut. The protrusion and residual and loose vertebral pulp tissue within the intervertebral space were further removed.

### 2.3 Medication regimen

Group A: The patients were administered 40 mg parecoxib through an intravenous injection twice a day from 2 days before the surgery. The drug was continuously administered till the fifth day after the surgery. Group B: The patients received 200 mg celecoxib through oral administration twice a day from 2 days before the surgery. The drug was continuously administered till the fifth day after the surgery. Group C: The patients received a buprenorphine transdermal patch (5 mg) on one-third of the lateral upper arm (right side or left side). If a skin defect or a larger scar was detected on the bilateral upper arm, the buprenorphine transdermal patch could be attached to the upper chest. The patch was not removed during the surgery and conserved till the fifth day after the surgery.

### 2.4 Observation indexes

The pain condition of each patient was recorded at the following time points: the night before surgery, postoperative day 1, postoperative day 3, and postoperative day 5. The VAS score VAS was applied to evaluate the degree of pain in patients: 0 to 2 scores indicated excellent; 3 to 5 scores indicated good; 6 to 8 scores indicated fair; and 9 to 10 scores indicated poor. Meanwhile, the occurrence of adverse effects and the condition in which the patient was administered tramadol hydrochloride for uncontrolled pain were also recorded. The degree of patient satisfaction was evaluated according to the satisfaction questionnaire.

### 2.5 Statistical analysis

Statistical analysis was performed using the Software of Statistical Program for Social Sciences 20.0 (SPSS 20.0 for windows, © SPSS Inc.). Continuous variables were expressed as mean ± standard deviation. Categorical data were presented as counts and percentages. The Student *t* test, chi-square test, or Fisher's exact test was used to compare the 2 groups, as appropriate. One-way analysis of variance was applied to compare the data among different groups. A *P* value less than .05 was considered statistically significant.

## 3 Results

### 3.1 Comparison of analgesic effect (VAS score) between the 3 groups

The VAS scores of the patients at each time point were compared among the groups. The results are listed in Table 2. The VAS scores of the patients in the 3 groups were significantly improved after drug administration compared with the VAS scores before drug administration. No significant differences in VAS scores on postoperative days 3 and 5 were observed in each group (*P* > .05). The VAS scores of Groups A and B were superior to those of patients in Group B on the night before surgery and postoperative day 1. The results showed a significant difference (*P* < .05). However, no significant difference in VAS scores at the aforementioned time points was found between Groups A and C (*P* > .05).

**Table 2:**
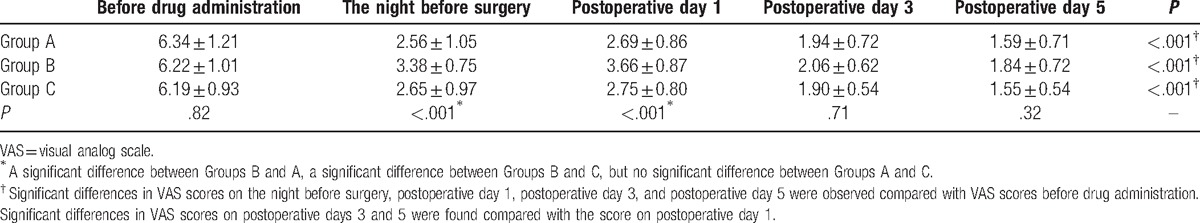
Comparisons of analgesic effect (VAS score) between the 3 groups at different time points.

### 3.2 Comparisons of adverse effects between the 3 groups

The occurrence rate of nausea and vomiting, drowsiness, and postoperative delirium was not significantly different between the 3 groups (*P* > .05). Two patients in Group C suffered from local skin allergy and pruritus, but without a significant difference compared with the other 2 groups (*P* > .05). No patient suffered from severe complications, such as peptic ulcer and respiratory inhibition, and so on, in the 3 groups. If the patient suffered from 2 or more than 2 symptoms during drug administration, the condition was considered as just 1 patient who had adverse effects. The results are listed in Table 3.

**Table 3:**
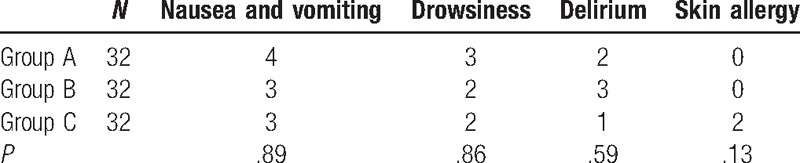
Comparisons of adverse effects between the 3 groups.

### 3.3 Comparisons of the degree of patient satisfaction between the 3 groups

The degree of patient satisfaction in Group C was higher than that in Groups A and B, with a significant difference (*P* < .05). No significant difference in the degree of patient satisfaction was observed between Groups A and B (*P* > .05). The results are listed in Table 4.

**Table 4:**
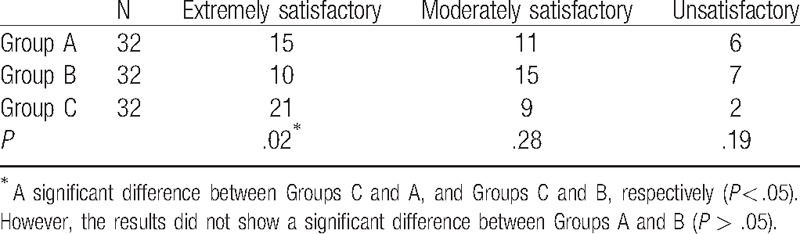
Comparisons of the degree of patient satisfaction between the 3 groups.

### 3.4 Comparison of postoperative additional dosage of tramadol hydrochloride in the 3 groups

No patient in Group A had a postoperative additional dosage of tramadol hydrochloride. Two cases in Group B and 1 case in Group C had a postoperative additional dosage of tramadol hydrochloride (100 mg, once, intramuscular injection).

## 4 Discussion

Simple discectomy is one of the effective methods to treat LDH. Whether the surgery is successful has a direct relationship with the postoperative analgesic effect. A reasonable analgesic effect not only can reduce the physical and psychological pain in patients, but also is beneficial to the functional recovery at an early stage. Meanwhile, good analgesia can reduce cardiovascular complications and promote early rehabilitation. Opioid analgesic drugs, such as morphine, pethidine, fentanyl, and tramadol, are commonly used drugs for postoperative analgesia in clinical practice. These drugs have severe adverse effects, including nausea and vomiting, dizziness, headache, drowsiness, urinary retention, skin itching, respiratory depression, easy addiction, and so on. Meanwhile, the occurrence rate of drug adverse effects is higher in the older population or patients with poor constitution. Nonsteroidal antiinflammatory drugs, such as ibuprofen, indomethacin, celecoxib, and so on, are another kind of analgesic drugs commonly used in clinical practice.^[10]^ They can exert adverse effects to different degrees on the digestive tract, platelets, and kidneys of patients.^[11,12]^

A buprenorphine transdermal patch is a kind of synthetic opioid analgesic. It is also a partial agonist for the μ-opioid receptor and exerts an antagonistic effect for κ and δ receptors. The analgesia intensity of equivalent dose is 75 to 100 times that of morphine. It also has good skin penetration and a lasting analgesic effect. A buprenorphine transdermal patch has no obvious gastrointestinal reaction and addiction and is easy to use. A buprenorphine transdermal patch has entered into the Chinese market since July 2013.^[13–15]^ Privitera and Guzzetta^[16]^ applied a buprenorphine transdermal patch for the postoperative pain management of elderly patients who underwent orthopedic surgery. The results of this study found that the buprenorphine transdermal patch could significantly reduce the pain score, improve the sleep quality, decrease adverse effects and additional dosage of analgesic drugs, and enhance the degree of patient satisfaction. Setti et al^[17]^ found that a large dose of buprenorphine transdermal patch could effectively reduce postoperative pain after gynecological surgery, and further reduce the additional dose of analgesic drugs during the early postoperative period.

A buprenorphine transdermal patch (preemptive analgesia regimen) was applied in this study to control the perioperative pain of patients who underwent simple LDH discectomy. This study found that the analgesic effect of buprenorphine transdermal patch was better compared with the conventional analgesic regimen of parecoxib intravenous injection and oral celecoxib; it could effectively control the perioperative pain. Moreover, the application of buprenorphine transdermal patch could not increase the related adverse effect of perioperative analgesia compared with the conventional analgesic regimen of parecoxib intravenous injection and oral celecoxib. The degree of patient satisfaction in the buprenorphine transdermal patch group was significantly higher (*P* < .05), and the VAS score was not significantly higher, compared with that in the other 2 groups. This was because of the advantages of the drug administration approach. The patients in the parecoxib and celecoxib groups were administered the drugs twice a day, but the patients in the buprenorphine group received the transdermal patch on the local skin, to achieve the objective of continuous administration for more convenience to the patients, avoid oral and intravenous drugs, and provide psychological comfort to the patients. This study indicated that the buprenorphine transdermal patch (preemptive analgesia regimen) could exert the analgesic effect on patients who underwent simple discectomy during the perioperative period, which was beneficial for patients to sustain postoperative physiological and psychological states, and promote functional rehabilitation.

This study had some limitations. On the one hand, the sample size in this study was limited. Therefore, the findings could not relate to the real situation of preemptive analgesia regimen of the buprenorphine transdermal patch on a large scale. On the other hand, the 3 groups in this study involved different modes of drug administration: intravenous, oral, and transdermal. Hence, a double-blind design could not be achieved. Moreover, different pathways of drug administration might exert a certain degree of influence on the psychological status of the patients, resulting in the errors in study results. Also, the VAS score is also influenced by the degree of anxiety in patients, the expectancy value of the treatment, and other factors, although it is recognized as the most sensitive and reliable pain assessment methods in clinical practice. Therefore, large-sample double-blind controlled trials should be conducted in the future. Also, more objective and reliable pain assessment methods should be used to obtain more accurate and reliable data and conclusions.
